# Soya-cerebroside, an extract of Cordyceps militaris, suppresses monocyte migration and prevents cartilage degradation in inflammatory animal models

**DOI:** 10.1038/srep43205

**Published:** 2017-02-22

**Authors:** Shan-Chi Liu, Ching-Peng Chiu, Chun-Hao Tsai, Chun-Yin Hung, Te-Mao Li, Yang-Chang Wu, Chih-Hsin Tang

**Affiliations:** 1 Graduate Institute of Basic Medical Science, China Medical University, Taichung, Taiwan; 2 Department of Orthopedic Surgery, Taichung Veterans General Hospital, Taichung, Taiwan; 3 Graduate Institute of Natural Products, College of Pharmacy, Kaohsiung Medical University, Kaohsiung, Taiwan; 4 Graduate Institute of Clinical Medical Science, China Medical University, Taichung, Taiwan; 5 Department of Orthopedic Surgery, China Medical University Hospital, Taichung, Taiwan; 6 Department of Orthopaedic Surgery, China Medical University Beigang Hospital, Yun-Lin County, Taiwan; 7 School of Chinese Medicine, China Medical University, Taichung, Taiwan; 8 School of Pharmacy, College of Pharmacy, China Medical University, Taichung, Taiwan; 9 Research Center for Chinese Herbal Medicine, China Medical University, Taichung, Taiwan; 10 Chinese Medicine Research and Development Center, China Medical University Hospital, Taiwan; 11 Center of Molecular Medicine, China Medical University Hospital, Taichung, Taiwan; 12 Department of Pharmacology, School of Medicine, China Medical University, Taichung, Taiwan; 13 Department of Biotechnology, College of Health Science, Asia University, Taichung, Taiwan

## Abstract

Pathophysiological events that modulate the progression of structural changes in osteoarthritis (OA) include the secretion of inflammatory molecules, such as proinflammatory cytokines. Interleukin-1beta (IL-1β) is the prototypical inflammatory cytokine that activates OA synovial cells to release cytokines and chemokines in support of the inflammatory response. The monocyte chemoattractant protein-1 (MCP-1/CCL2) is one of the key chemokines that regulate migration and infiltration of monocytes in response to inflammation. We show in this study that IL-1β-induced MCP-1 expression and monocyte migration in OA synovial fibroblasts (OASFs) is effectively inhibited by soya-cerebroside, an extract of *Cordyceps militaris*. We found that soya-cerebroside up-regulated of microRNA (miR)-432 expression via inhibiting AMPK and AKT signaling pathways in OASFs. Soya-cerebroside also effectively decreased monocyte infiltration and prevented cartilage degradation in a rat inflammatory model. Our findings are the first to demonstrate that soya-cerebroside inhibits monocyte/macrophage infiltration into synoviocytes, attenuating synovial inflammation and preventing cartilage damage by reducing MCP-1 expression *in vitro* and *in vivo*. Taken together, we suggest a novel therapeutic strategy based on the use of soya-cerebroside for the management of OA.

Osteoarthritis (OA) is a degenerative joint disease characterized by slow progressive cartilage degradation and synovial inflammation^1^. Biochemical mediators found in OA synovial fibroblasts (OASFs) that affect the cellular functions of multiple joint tissues include cytokines, chemokines, growth factors, and matrix metalloproteinases (MMPs)^2^. Although the pathogenesis of OA is complicated and remains poorly understood, leukocyte trafficking from the vascular lumen to sites of inflammatory stimuli is essential for effective immune surveillance^3^,^4^. Activation and accumulation of mononuclear leukocytes to inflammatory sites is regulated by chemokines, such as monocyte chemoattractant protein-1 (MCP-1)^5^.

MCP-1, also known as chemokine ligand 2 (CCL2), belongs to the CC chemokine family. MCP-1 is chemotactic for monocyte/macrophages and activated T cells^6^. Increased concentrations of MCP-1 are detected in the blood, synovial fluid and synovial tissue in patients with OA^7^. Injection of MCP-1 into rabbit joints causes marked macrophage migration into the affected joint^8^. In animals suffering experimentally also indicated that over-expressed MCP-1 could induce synovitis^9^. It has also been reported that treatment with an MCP-1 antagonist prior to disease onset prevents the development of arthritis in a mouse model^10^. These data are supported by further research suggesting that MCP-1 may play a crucial role as a proinflammatory agent during OA pathogenesis^11^,^12^,^13^.

MicroRNAs (miRNAs) are evolutionarily conserved, small, non-coding RNAs (19–25 nucleotides in length) that post-transcriptionally modulate the expression of downstream target genes by repressing translation or accelerating mRNA degradation^14^,^15^. MiRNAs are increasingly implicated in cartilage homeostasis and OA pathogenesis, especially in chondrocytes expression of genes encoding catabolic factors, such as matrix metalloproteinases (MMPs) and aggrecanase-1 (ADAMTS4)^16^. Much evidence indicates that miR-432 expression is reduced in various tumors, such as hepatocellular carcinoma, cervical and ovarian cancer^17^,^18^. Recent findings show that miR-432 functions as a tumor-suppressive miRNA in hepatocellular carcinoma cells and may represent a prognostic parameter and therapeutic target for lung adenocarcinoma^19^. However, the exact etiological mechanism of miR-432 in monocyte infiltration and OA pathogenesis is largely unknown.

*Cordyceps militaris*, an entomopathogenic fungus belonging to the Ascomycetes class, has been widely used as an herbal medicine for inflammatory diseases in humans^20^. Soya-cerebroside, an extract of *C. militaris*, showed significant anti-inflammatory activity by inhibiting inducible nitric oxide synthase (iNOS) and cyclooxygenase-2 (COX-2) expression in LPS-stimulated RAW264.7 cells^21^. However, the anti-inflammatory mechanism of soya-cerebroside in OA has not been completely elucidated. In the present study, we demonstrate how soya-cerebroside attenuates IL-1β-induced MCP-1 expression and promotes monocyte migration by up-regulating miR-432 expression via AMPK and AKT signaling pathways, as well as abolish macrophage infiltration and protect cartilage in inflammatory animal model. Our results show that soya-cerebroside is a potential therapeutic candidate for OA disease.

## Results

### Soya-cerebroside attenuates IL-1β-induced MCP-1 expression and monocyte migration

**Figure 1:**
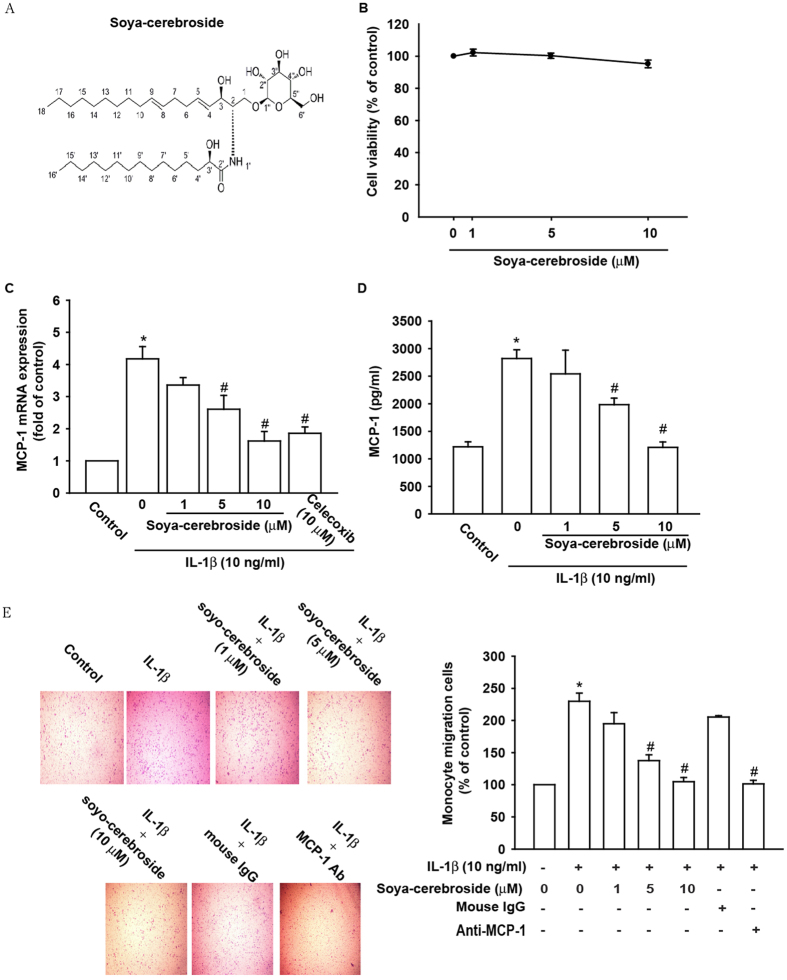
Soya-cerebroside downregulates MCP-1 expressions in OASFs and inhibited monocyte migration. (**A**) Soya-cerebroside chemical structure. (**B**) OASFs were treated for 24 h with soya-cerebroside at indicated concentrations. Cell viability was determined by MTT assay. (**C**) OASFs were treated with soya-cerebroside or celecoxib (10 μM) in the presence or absence of IL-1β (10 ng/mL) for 24 h. MCP-1 mRNA expression was tested by qPCR. (**D**) OASFs were treated with soya-cerebroside in the presence or absence of IL-1β (10 ng/mL) for 24 h. MCP-1 expression was tested by ELISA assay (**E**) Conditioned media were obtained by incubating the cells of IL-1β for 24 h or pretreating the cells with various concentrations of soya-cerebroside for 30 min followed by stimulation with IL-1β for 24 h. Monocyte (THP-1) migration was assessed by an *in vitro* chemotaxis assay. Similar studies were performed after the conditioned media were pretreated with mouse anti-human MCP-1 or mouse IgG for 30 min. The results are expressed as the mean ± SEM of triplicate samples. *p < 0.05 as compared with control group. ^#^p < 0.05 as compared to the IL-1β-treated group.

Studies indicated that soya-cerebroside has anti-inflammatory effects^21^. We hypothesized that it might decrease monocyte migration and inhibit inflammatory responses in OASFs. Cells were cultured with different concentrations of soya-cerebroside to evaluate the effect on cell viability. Findings revealed that treatment with soya-cerebroside dose-dependently inhibited the IL-1β-induced MCP-1 mRNA and protein secretion levels but did not influence viability of OASFs (Fig. 1B–D). Celecoxib is a selective COX-2 inhibitor^22^ which was used as the reference compound. We also found celecoxib (10 μM) inhibited IL-1β-induced MCP-1 expression (Fig. 1C). Furthermore, soya-cerebroside treatment significantly inhibited IL-1β promotion of monocyte migration (MCP-1-Ab was used as positive control) (Fig. 1E). These data suggest that soya-cerebroside eliminated IL-1β promotion of MCP-1 expression and monocyte migration in human synovial fibroblasts.

### Inhibition of MCP-1 expression and monocyte migration by soya-cerebroside via upregulation of miR-432 expression in OASFs

**Figure 2:**
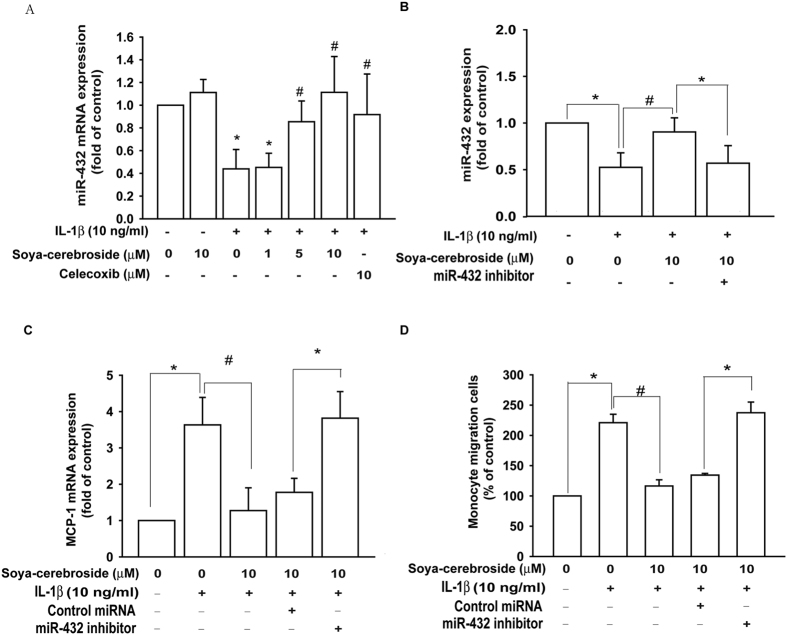
Soya-cerebroside inhibits MCP-1 expression and monocyte migration by upregulating miR-432 expression in OASFs. (**A**) OASFs were treated with soya-cerebroside or celecoxib (10 μM) in the presence or absence of IL-1β (10 ng/mL) for 24 h; miR-432 expression was examined by qPCR. (**B**) OASFs were transfected with the miR-432 inhibitor for 24 h prior to treatment with soya-cerebroside (10 μM), followed by IL-1β stimulation. MiR-432 expression was examined by qPCR. (**C**) OASFs were transfected with the miR-432 inhibitor or control miRNA for 24 h prior to treatment with soya-cerebroside (10 μM), followed by IL-1β stimulation. MCP-1 expression was examined by qPCR. (**D**) OASFs treated as explained in C; monocyte migration was examined by *in vitro* chemotaxis assay *p < 0.05 as compared with the control group. ^#^p < 0.05 as compared to the IL-1β-treated group.

Several miRNAs have been identified as showing differential expression patterns between OA and normal cartilage, and their postulated functions relate to inflammatory and catabolic changes, as well as angiogenesis, in OA^23^,^24^. However, the precise roles and mechanisms of miRNAs in OA remain far from clear. We used a customized miRNome microRNA Profilers Kit containing 384 human miRNAs and the expression of approximately 17 miRNAs were down-regulated by IL-1β. Soya-cerebroside treatment significantly rescued miR-432 levels compared with those in the IL-1β treated group (Supporting Information Fig. S1). To confirm this finding, we directly applied IL-1β to OASFs and found miR-432 expression was downregulated by approximately 2–2.5-fold. The decreased miR-432 levels in IL-1β-treated cells were significantly reversed by soya-cerebroside treatment in a dose-dependent way (1–10 μM). However, soya-cerebroside didn’t affect basal level of miR-432. Furthermore, celecoxib treatment also reversed IL-1β-decreased miR-432 expression in OASFs (Fig. 2A). To further determine if soya-cerebroside-mediated inhibition of MCP-1 expression was due to the regulation of miR-432 levels, a miR-432 inhibitor was used. Transfection with the miR-432 inhibitor diminished soya-cerebroside-induced miR-432 expression, leading to enhanced MCP-1 production (Fig. 2B and C). Similar results were observed with regard to monocyte migration. In addition, treatment with the miR-432 inhibitor prevented the inhibitory effects of soya-cerebroside on IL-1β-induced migration of monocytes (Fig. 2D). Transfection with control miRNA (negative control) didn’t affect soya-cerebroside-attenuated MCP-1 production and monocyte migration (Fig. 2C and D). These findings suggest that soya-cerebroside inhibited MCP-1 expression and monocyte migration by upregulating miR-432 expression in OASFs.

### Soya-cerebroside inhibits SP1 expression via upregulation of miR-432

**Figure 3:**
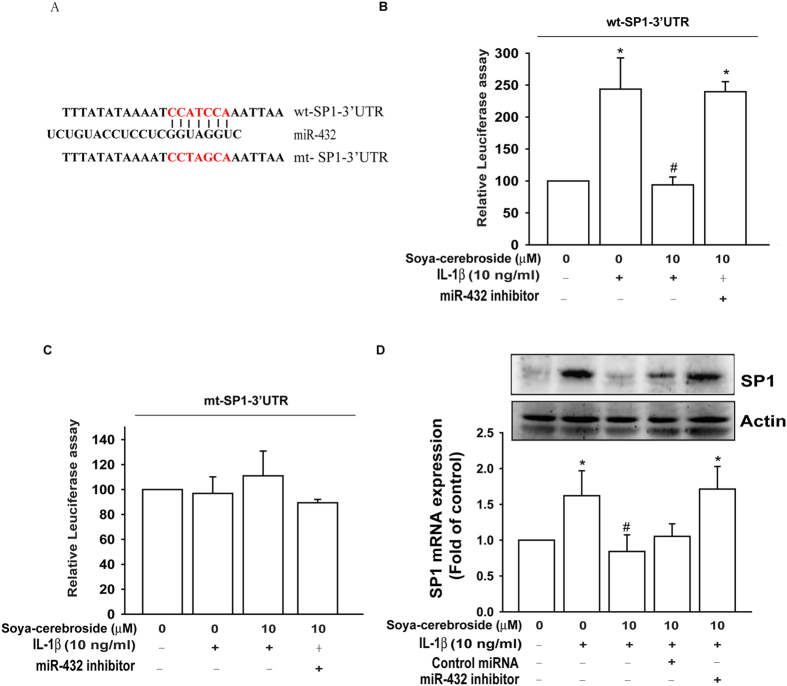
Soya-cerebroside inhibits SP1 expression via miR-432 regulation. (**A**) Schematic 3′UTR representation of the human SP1 containing the miR-432 binding site. (**B** and **C**) OASFs were co-transfected with a miR-432 inhibitor or control miRNA and wt-SP1-3′-UTR or mt-SP1-3′-UTR plasmid for 24 h prior to treatment with soya-cerebroside (10 μM), followed by IL-1β stimulation; relative luciferase/renilla activities were measured, as described in the Methods section. (**D**) OASFs were transfected with the miR-432 inhibitor or control miRNA prior to treatment with soya-cerebroside (10 μM), followed by IL-1β stimulation. The cells were collected and SP1 expression was examined by qPCR and western blot assay. Results are expressed as the percentage of control and presented as the mean ± S.E.M. (n = 3). Each blot is representative of three independent experiments. *p < 0.05 as compared with baseline. ^#^p < 0.05 as compared the IL-1β-treated group.

To identify the miR-432 mechanism involved in MCP-1 expression and monocyte migration, we searched for downstream genes using a bioinformative screening analysis of miRNA target databases: TargetScan, MicroCosm, and miRanda. The overlapping of these three databases between predicted targets of miR-432 identified SP1 as the most probable target. We used luciferase reporter vectors harboring wild-type 3′UTR of the SP1 mRNA (wt-SP1-3′UTR) and vectors containing mismatches in the predicted miR-432 binding site (mt-SP1-3′UTR) to learn whether miR-432 regulates the SP1 3′UTR (Fig. 3A). As shown in Fig. 3B and C, treatment with soya-cerebroside attenuated IL-1β-increased luciferase activity in the wt-SP1-3′UTR plasmid but not in the mt-SP1-3′UTR plasmid. These results were reversed the miRNA-432 inhibitor (Fig. 3B and C). Soya-cerebroside also attenuated IL-1β-induced SP1 mRNA and protein expression. Transfection with the miR-432 inhibitor but not control miRNA reversed inhibitory effects of soya-cerebroside on IL-1β-induced SP1 mRNA and protein expression (Fig. 3D). Thus, soya-cerebroside upregulated miR-432 by directly repressing SP1 expression via binding to the 3′UTR of the human *sp1* gene.

### AMPK/AKT signaling pathways are involved in soya-cerebroside-mediated miR-432 expression

**Figure 4:**
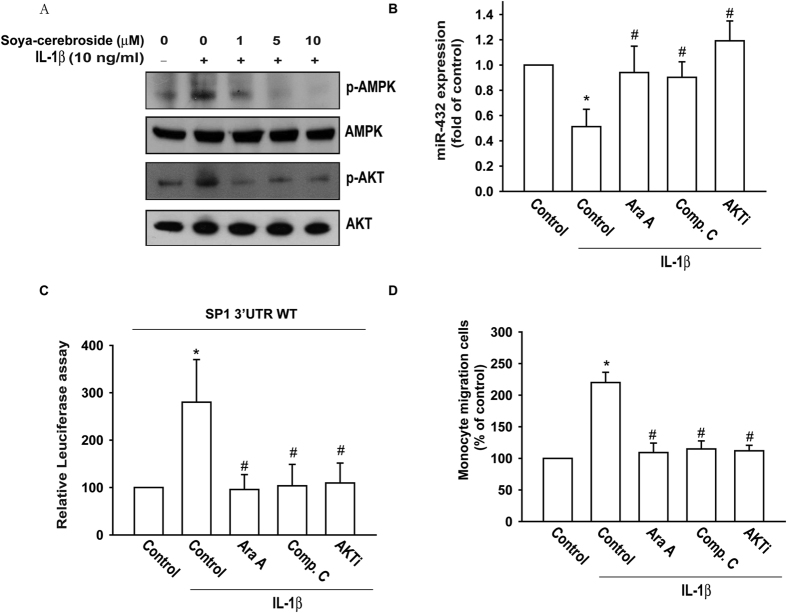
AMPK/AKT signaling pathways are involved in soya-cerebroside mediated miR-432 expression. (**A**) OASFs were treated with various soya-cerebroside concentrations in the presence or absence of IL-1β for 1 h, cells were collected, and AMPK and AKT phosphorylation was examined by Western blot. (**B**) OASFs were pretreated with Ara A, Compound C, and AKTi for 30 min followed by stimulation with IL-1β for 24 h; miR-432 expression was examined by qPCR. (**C**) OASFs were pretreated with Ara A, Compound C, and AKTi for 30 min, followed by stimulation with IL-1β for 24 h. The wt-SP1-3′-UTR relative luciferase/renilla activities were measured as described in the Methods section. (**D**) OASFs were treated as explained in C, monocyte migration was examined by *in vitro* chemotaxis assay *p < 0.05 as compared with the control group. ^#^p < 0.05 as compared to the IL-1β-treated group.

While it is recognized that the AMPK/AKT signaling pathways are associated with inflammation, increasing the production of inflammatory cytokines^25^, it remains unclear as to the mechanism by which miR-432 regulates AMPK/AKT signaling in OASFs after soya-cerebroside treatment. IL-1β treatment of OASFs induced phosphorylation of AMPK and AKT (Fig. 4A). In addition, soya-cerebroside dose-dependently inhibited the IL-1β-mediated phosphorylation of AMPK and AKT (Fig. 4A). Furthermore, treatment with an AMPK inhibitor (Ara A and Compound C) and an AKT inhibitor reversed IL-1β-inhibited miR-432 expression and also diminished IL-1β-induced wt-SP1-3′-UTR luciferase activity (Fig. 4B and C) and monocyte migration (Fig. 4D).

### Soya-cerebroside inhibits knee joint inflammation in inflammatory animal models

**Figure 5:**
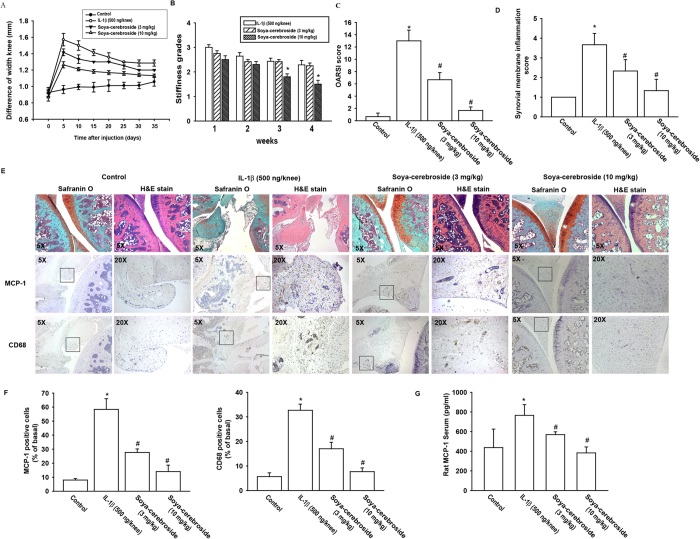
Effects of soya-cerebroside on monocyte infiltration and prevented cartilage damage in inflammatory animal model. Shaved right knee joints of all rats (n = 8 per group) were injected intra-articularly with either 50 μl (500 ng) of recombinant human IL-1β (rhIL-1β) or saline as a negative control. After initial injection of rhIL-1β (Day 0), rats were divided into 3 groups and administered IP injections of saline, soya-cerebroside (3 mg/kg), or soya-cerebroside (10 mg/kg) daily for 35 days. (**A**) Joint swelling was assessed by measuring knee diameters on the days indicated after rhIL-1β induction. (**B**) Stiffness was rated by motility of knee joints and graded on a scale of 0–4. (**C**) OARSI score of articular cartilage sections stained with Safranin-O was calculated. (**D**) Synovial membrane inflammation score. Magnified area of synovium used to generate synovial inflammation score in all samples. Scoring was done in H&E stained slides. (**E**) Photomicrographs of knee joint sections from different groups stained with H&E or Safranin O (magnification 5X) and immunostained with MCP-1 or CD68 (Left panel of each group original magnification 5X and Right panel of each group original magnification 20X). (**F**) The quantitative data of MCP-1 and CD68 postive cells are presented. (**G**) Serum MCP-1 on Day 35 was measured by ELISA assay. *p < 0.05 as compared to control. ^#^p < 0.05 as compared to the IL-1β-treated group.

**Figure 6:**
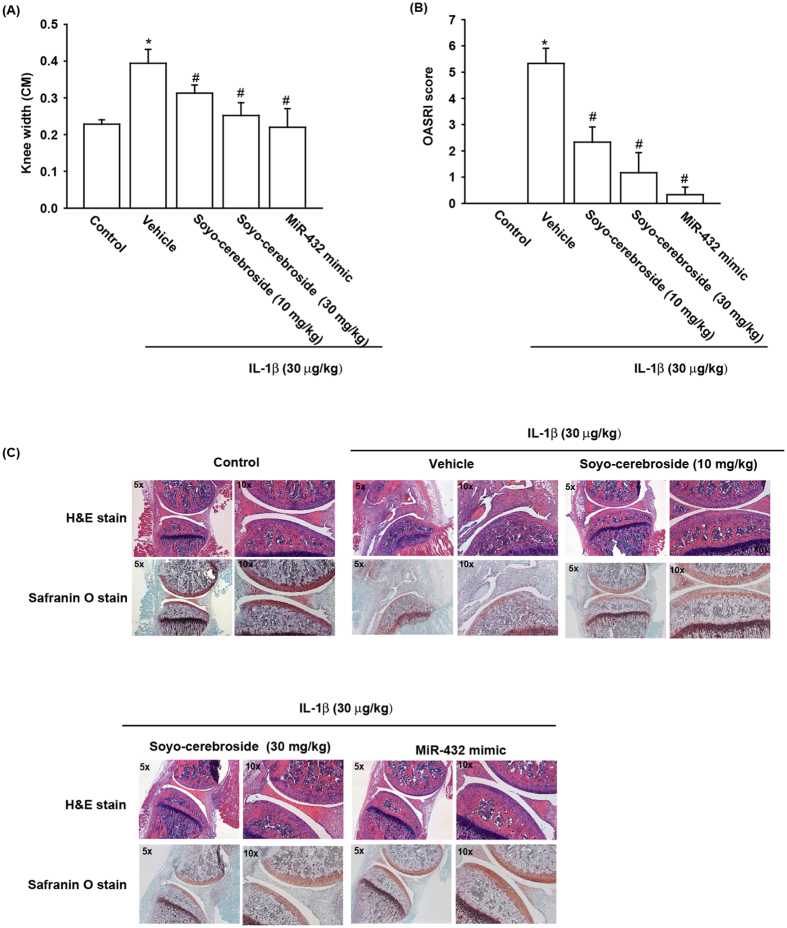
Soya-cerebroside prevented cartilage damage in inflammatory mice model. Mice were given an intraarticular injection of saline, Soya-cerebroside or miR-432 mimic, which was followed 24 hours later with an intraarticular of 20 μl (30 μg/kg) of recombinant human IL-1β (rhIL-1β). Joints were harvested and examined after 7 days (n = 8 per group). (**A**) Stiffness was rated by motility of knee joints and graded on a scale of 0–4. (**B**) OARSI score of articular cartilage sections stained with Safranin-O was calculated. (**C**) Photomicrographs of knee joint sections from different groups stained with H&E or Safranin O (Left panel of each group original magnification 5X and Right panel of each group original magnification 10X). *p < 0.05 as compared to control. ^#^p < 0.05 as compared to the IL-1β-treated group.

To investigate the effect of soya-cerebroside on IL-1β-induced joint inflammation, we intra-articularly injected recombinant human IL-1β (rhIL-1β) into the rat knee joint to induce synovial inflammation. Knee joint inflammation was monitored by assessment of joint swelling. The results show that soya-cerebroside significantly attenuated knee widths compared with knees in the IL-1β-induced group, as reflected by stiffness data (Fig. 5A and B). The OARSI score and synovial membrane inflammation score showed that more than 75% cartilage destruction and synovial inflammation in IL-1β-induced inflammatory rat. In contrast, soya-cerebroside effectively reversed the cartilage damage and synovial inflammation in dose-dependent way (Fig. 5C–E). Immunohistochemical staining of the knee revealed that the number of cells positive for MCP-1 and CD68 (a marker for monocyte/macrophages) were significantly increased in IL-1β induced group but markedly diminished in rats treated with soya-cerebroside. Figure 5F showed the quantitative data of MCP-1 and CD68 positive cells. In addition, MCP-1 serum levels were significantly increased in the IL-1β-induced (Fig. 5G), whereas the soya-cerebroside-treated animals had lower MCP-1 levels than IL-1β-induced group (Fig. 5G). Similar results were obtained when analyzed in an inflammatory mice model: Intraarticular injection of miR-432 mimic or soya-cerebroside inhibited the IL-1β-induced knee widths and cartilage damage in inflammatory mice model (Fig. 6). These results suggest that soya-cerebroside effectively decreased monocyte infiltration to prevent cartilage erosion *in vivo*.

## Discussion

Although the pathogenesis of OA is complicated and remains poorly understood, leukocyte infiltration to sites of inflammatory stimuli is essential for effective immune surveillance^3^,^4^. This suggests that synovial inflammation plays a crucial role in OA pathophysiology^26^. Infiltration of monocytes into inflammatory sites is mainly regulated by MCP-1, which is expressed on synoviocytes. Inflammatory cytokines such as CTGF can increase the expression of MCP-1 in OASFs through NF-κB- and AP-1- mediated mechanisms^27^; further, they recruit leukocytes, which migrate into target tissues via MCP-1 injection *in vivo*^8^ and induce synovitis^9^. Therefore, inhibition of MCP-1 expression and monocyte migration to reduced inflammation may be an effective therapeutic approach to inflammatory arthritis^12^. Our study found that soya-cerebroside attenuates IL-1β-induced MCP-1 expression and the ability of monocytes to migrate, through up-regulation of miR-432 expression and inhibition of AMPK and AKT signaling pathways in OASFs, while eliminating cartilage degradation and monocyte infiltration in the IL-1β-induced rat inflammatory model. We also used an inflammatory mice model to confirm the effect of soya-cerebroside, although miR-432 does not exist in rat. Similar results were obtained: Intraarticular injection of miR-432 mimic or soya-cerebroside could ameliorate the disease severity in the IL-1β induced mice. Our findings suggest that soya-cerebroside could be a potential therapeutic option in the treatment of OA.

Miller R. E. *et al*. has been indicated that Ccr2 (MCP-1 receptor) deficient mice exhibited cartilage degradation in surgically induced OA model. Furthermore, the macrophage infiltration was not found in Ccr2-null mice^28^. Although, MCP-1 increased the resident macrophages (CX3CR1^hi^ CCR2^−^Gr1^−^) accumulation to increase numbers of osteoclasts leading to enhance bone destruction in the joints of CCR2 deficient mice with CIA (rheumatoid arthritis animal model)^29^. In the present study, we found soya-cerebroside attenuates IL-1β-induced MCP-1 expression and monocyte infiltration in inflammatory animal model. Therefore, MCP-1 may play different role in OA, RA, and inflammatory animal models.

In recent years, miRNA findings have improved our understanding of the regulation of physiological and pathological processes. Significant abnormal miRNA expression profiles have been reported in OA^30^. MiR-146, miR-155 and miR-203 regulate arthritic inflammatory response and joint destruction^31^. We screened 384 miRNAs from a customized miRNA array and discovered that soya-cerebroside treatment significantly reverses IL-1β downregulation of miR-432 levels. MiR-432 functions as a tumor-suppressive miRNA in hepatocellular carcinoma cells and may represent a prognostic parameter and therapeutic target for lung adenocarcinoma^19^. However, no studies have previously explored the function of miR-432 in OA. In this study, qPCR analysis confirmed that miR-432 was starkly downregulated in IL-1β-treated cells. Treatment with soya-cerebroside inhibited MCP-1 expression and monocyte migration via upregulation of miR-432 expression in OASFs. The promoter region of human MCP-1 contains SP1 binding sites^32^. Our study found that miR-432 directly represses SP1 mRNA and protein expression through binding to the 3′-UTR of the human *sp1* gene, thereby showing that miR-432 can negatively regulate MCP-1 production as well as monocyte migration.

Evidence indicates that AMPK and AKT signaling pathways play an important role in the production of proinflammatory cytokines^25^. Here, we report that IL-1β incubation of OASFs enhanced phosphorylation of AMPK and AKT. Pretreatment with soya-cerebroside dose-dependently inhibited IL-1β-mediated phosphorylation of AMPK and AKT. Therefore, AMPK and AKT signaling pathways mediate IL-1β-induced MCP-1 expression and monocyte migration. Furthermore, treatment with AMPK or AKT inhibitors reversed IL-1β-inhibited miR-432 expression and diminished IL-1β-promoted wt-SP1-3′-UTR luciferase activity, implying that AMPK and AKT play a critical role in IL-1β-diminished miR-432 expression.

Nonsteroidal anti-inflammatory drugs (NSAIDs), are the most prescribed medications for treating conditions such as OA. However, the side effects of long-term uses NSAIDs were described^33^. Phytochemicals can modulate inflammatory response and treat many diseases such as OA^34^. Soya-cerebroside showed anti-inflammatory activity by inhibiting iNOS and COX-2 expression in LPS-stimulated RAW264.7 cells^21^. This study identified the anti-inflammation effect in soya-cerebroside of human synovial fibroblasts and examined whether soya-cerebroside prevents the expression of MCP-1, a key chemokine, in IL-1β-stimulated OASFs. The anti-inflammatory effect of soya-cerebroside attenuated IL-1β-induced MCP-1 expression and the migration ability of monocytes via the up-regulation of miR-432 expression and inhibiting AMPK and AKT signaling pathways in OASFs. Soya-cerebroside also effectively inhibited monocyte infiltration and prevented cartilage degradation in the IL-1β-induced rat inflammatory model. Soya-cerebroside could therefore be a potential therapeutic option in the treatment of OA.

## Materials and Methods

### Materials

Soya-cerebrosides (Fig. 1A) of >98% purity were synthesized at the College of Pharmacy, School of Pharmacy, China Medical University, Taichung, Taiwan (The detail synthesis procedures and purified quality was provided as described previously^21^). Soya-cerebroside was dissolved in DMSO for intraperitoneal (IP) injection. A miR-432 inhibitor, Control miRNA and TRIzol were from Life Technologies (Carlsbad, CA). Rabbit polyclonal antibody specific for MCP-1, MCP-1 enzyme-linked immunosorbent assay (ELISA) kit and human recombinant IL-1β were purchased from R&D Systems (Minneapolis, MN, USA), anti-phospho AMPK (Thr172) and phospho AKT (Ser473) from Cell Signaling (Danvers, MA, USA). Mouse monoclonal CD68 was purchased from Novus (Novus, Littleton, USA) and Ara A (ATP mimetic), Compound C (AMPK kinase inhibitor), and AKTi (AKT1/2 kinase inhibitor) from Enzo Biochem, Inc. (Enzo, New York, NY). Anti-AMPK, anti-AKT, and anti-mouse and anti-rabbit conjugated horseradish peroxidase (HRP) antibodies were from Santa Cruz (Santa Cruz, CA, USA). All other chemicals were purchased from Sigma-Aldrich (St. Louis, MO, USA).

### Cell culture

Synovial tissue from the suprapatellar pouch of the knee were obtained from 20 patients (mean age 76.3 years) diagnosed as having stage IV OA. The cells were cultured in DMEM medium supplemented (Gibco, USA) with 10% fetal bovine serum (FBS) (Gibco), 50 units/mL penicillin (Gibco), and 50 μg/mL streptomycin (Gibco) as previously described^35^. The Institutional Review Board of China Medical University Hospital approved the protocol, and all the methods were carried out in accordance with the guidelines and regulations by the Institutional Review Board of the China Medical University Hospital. Informed written consent was obtained from all patients. THP-1, a human leukemia cell line of monocyte/macrophage lineage, was obtained from American Type Culture Collection (Manassas, VA, USA) and grown in RPMI-1640 medium with 10% FBS^36^,^37^.

### MTT assay

For measurement of cell viability, cells were seeded onto 96-well culture plates at 8 × 10^4^ cells per well, cultured overnight in Dulbecco’s modified Eagle’s medium containing 10% fetal bovine serum and then treated with soya-cerebroside for 24 h. Cell viability was determined by 3-(4,5-cimethylthiazol-2-yl)-2,5-diphenyl tetrazolium bromide (MTT) assay^38^.

### Real-time quantitative PCR of mRNA and miRNA

Total RNA was extracted from human synovial fibroblasts by TRIzol; reverse transcription used 1 μg of total RNA transcribed into cDNA by oligo (dT) primers^39^. Real-time quantitative PCR (RT-qPCR) used Taqman^®^ One-Step RT-PCR Master Mix (Applied Biosystems, CA): two microliters of cDNA template were added to each 25-μL reaction with Taqman^®^ probes and sequence-specific primers. Sequences for target gene primers and probes were purchased commercially. Glyceraldehyde 3-phosphate dehydrogenase (GAPDH) served as an endogenous control to normalize expression data (Applied Biosystems); qPCR assays were carried out in triplicate in a StepOnePlus sequence detection system. The cycling conditions involved an initial 10-min polymerase activation at 95 °C, followed by 40 cycles at 95 °C for 15 s and 60 °C for 60 s, with the threshold set above the non-template control background and within the linear phase of target gene amplification, to calculate the cycle number at which the transcript was detected (denoted CT). For the miRNA assay, cDNA was synthesized from total RNA (100 ng) using the TaqMan MicroRNA Reverse Transcription Kit (Applied Biosystems). Reactants were incubated at 16 °C for 30 min, then 42 °C for 30 min, followed by inactivation at 85 °C for 5 min. Reactions were then incubated in 96-well plates at 50 °C for 2 min, 95 °C for 10 min, followed by 30 cycles of 95 °C for 15 s and 60 °C for 60 s with the StepOnePlus sequence detection system. Relative gene expression quantification used the endogenous control gene (U6). The threshold cycle (CT) was defined as the fractional cycle number at which fluorescence passed a fixed threshold, with the relative expression calculated by the comparative CT method.

### Measuring MCP-1 production

OASFs (2.5 × 10^4^) were pretreated with soya-cerebroside (0–10 μM) for 24 h before and during incubation with IL-1β (10 ng/mL) for another 24 h. After incubation, the medium was collected and assayed for MCP-1 using enzyme immunoassay kits.

### Western blot analysis

Lysates were prepared as described previously^40^,^41^, protein concentrations were determined using a BCA Protein Assay kit. The proteins were separated by SDS-polyacrylamide gel electrophoresis and transferred onto Immobilon polyvinyl difluoride membranes (Immobilon P, Millipore), which were blocked for 1 h at room temperature with 4% BSA. The expression of AMPK and Akt (Santa Cruz Biotechnology) was analyzed using specific antibodies. Immunoreactive proteins were visualized by enhanced chemiluminescence detection of secondary antibodies against mouse or rabbit antigens conjugated to horseradish peroxidase.

### *In vitro* chemotaxis assay

Conditioned media obtained from IL-1β-treated OASFs or pretreated with soya-cerebroside OASFs were placed in the lower compartment of Transwell cluster plates (Costa Corning, Cambridge, MA, USA) with two-compartment chambers separated by a polycarbonate membrane filter. THP-1 cells (5 × 10^4^ cells) were added to the upper chamber and incubated at 37 °C for 4 h, then fixed in 3.7% formaldehyde for 5 min and stained with 0.05% crystal violet in phosphate-buffered saline (PBS) for 15 min. Cells on the upper side of the filters were removed with cotton-tipped swabs and the filters were washed with PBS. Cells on the underside of the filters were examined and counted under a microscope. A mouse anti-human MCP-1 or mouse IgG was used in the chemotaxis assay media to determine the biological specificity of chemokines produced by the cells to stimulate monocyte migration.

### Plasmids and constructs

The wild-type and mutant SP-1 3′-UTRs were generated on the miR-432 target recognition sites (seed sequences). Both the wild-type and mutated 3′-UTRs of SP-1 were cloned into the pGL2 luciferase reporter vector using NheI and BglII restriction sites. The primer sequences used were defined as follows: SP-1 forward primer: GGGCTAGCTGAAAGTGAAACCAACATCCAGA, the reverse primer: GGAGATCTAAACAACACACTTACCCAAAACT, mutant SP-1 3′-UTR forward primer: TTTATATAAAATCCTAGCAAATTAAGTTTTGGGTAAGTGTG, mutant SP-1 3′-UTR reverse primer: CACACTTACCCAAAACTTAATTTGCTAGGATTTTATATAAA. All constructs were sequenced to verify that they contained the 3′-UTR inserts.

### Luciferase reporter assay

For reporter assays, OASFs were transiently transfected with pGL2 luciferase reporter vectors harboring wild-type or mutant target sequences together with miRNA mimics. Collected cells were lysed with reporter lysis buffer 24 h after transfection, and the ratio of Renilla to firefly luciferase activity was measured with the Dual-Luciferase^®^ Reporter Assay System (Promega Corp, Madison, WI, USA).

### Experimental rat IL-1β-induced inflammatory model

Experimental IL-1β-induced inflammatory model, which has relatively high synovial inflammation, was induced as previously described^42^,^43^. Male Sprague-Dawley rats (5 months old, 160 ± 20 g) were used in this study. Briefly, the hind legs of rat were shaved and prepared using routine aseptic techniques. All intra-articular injections were performed under ether anesthesia. The rats were given recombinant human IL-1β (rhIL-1β) by intraarticular injection to the right hind knee joint 50 μL (500 ng) on days 0 and 7 to elicit an inflammatory response. Following initial injection of IL-1β (Day 0), rats were administered intraperitoneal (IP) injections of saline, soya-cerebroside (3 mg/kg), or soya-cerebroside (10 mg/kg) daily for 35 days. The severity of arthritis in each knee was measured with a plethysmometer (Marsap, Mumbai, India). Stiffness was determined by changes in movement, swelling, and reddening of knees. Examination was performed by 2 independent observers who were blinded to the treatment groups. Rats were sacrificed and blood was collected by cardiac puncture on Day 35. All animal procedures were approved and performed in accordance with the guidelines of the Institutional Animal Care and Use Committee of China Medical University.

### Histological analysis and immunohistochemistry

The knee joints of the rats were removed and separated from the surrounding tissues. The lateral and medial sides of the femoral condyle and tibial plateau were decalcified in 10% EDTA at 4 °C for 2 weeks^38^, Serial sections (5 μm thick) were cutted from the medial side of knee joint and stained with Safranin-O/fast green and hematoxylin and eosin to examine the histopathological changes during the experimental period in all the groups treated with active compound under light microscope. The tissue sections were incubated with anti-MCP-1 or CD68 (1:200) primary antibody at 4 °C overnight and then incubated with secondary antibody (1:200) for 1 hr at room temperature. Finally, the sections were stained with diaminobenzidine.

The Osteoarthritis Research Society International (OARSI) scoring system, adapted for sagittal sections, was used to measure structural cartilage changes in the central weight bearing area of the medial tibial plateau in all samples^44^. In this system, the grade of damage from 0 to 6 is defined as the depth of progression of OA into the cartilage and the stage of damage is defined as the horizontal extent of cartilage involvement from 0 to 4. The final score is the combined value of grade and stage (score range 0–24). The grade of synovial membrane inflammation (Grade 0 = no changes, Grade 1 = > 3–4 lining cell layers or slight proliferation of subsynovial tissue, Grade 2 = > 3–4 lining cell layers and proliferation of subsynovial tissue, Grade 3 = > 4 lining cell layers and proliferation plus infiltration of subsynovial tissue with inflammatory cells, Grade 4 = > 4 lining cell layers and proliferation plus infiltration of subsynovial tissue with a large number of inflammatory cells). The examination was performed blindly by two observers and the scores were averaged to minimize observer bias.

### Experimental mice IL-1β-induced inflammatory model

SCID mice ages 4–6 weeks were purchased from National Laboratory Animal Centre in Taipei. Mice were intraarticular injection of saline, soya-cerebroside (10 mg/kg), soya-cerebroside (30 mg/kg) or mimic miRNA-432 [10 μg of miR-432 mixed with equal volume of Invivofectamine transfection reagent (Invitrogen, Carlsbad, CA)]. After 24 hours, mice were given an intraarticular injection of recombinant human IL-1β (rhIL-1β) (30 μg/kg) and were euthanized until to 7 days^45^. For OA grading, we used the OARSI histopathology scoring system for OA in mouse^46^, as shown in Supplementary Table S1.

### Statistical Analysis

Data are presented as the mean ± SD. Analysis of variance (ANOVA) and the unpaired two-tailed Student’s *t* test were used to compare differences between means. A p value of 0.05 or less was considered statistically significant.

## Additional Information

**How to cite this article**: Liu, S.-C. *et al*. Soya-cerebroside, an extract of Cordyceps militaris, suppresses monocyte migration and prevents cartilage degradation in inflammatory animal models. *Sci. Rep.* **7**, 43205; doi: 10.1038/srep43205 (2017).

**Publisher's note:** Springer Nature remains neutral with regard to jurisdictional claims in published maps and institutional affiliations.

## Supplementary Material

Supplementary Dataset 1
