# Supplementary material for: Soya-cerebroside, an extract of Cordyceps militaris, suppresses monocyte migration and prevents cartilage degradation in inflammatory animal models
Source: Sci Rep. 2017 Feb 22;7:43205. doi: 10.1038/srep43205 (PMC5320555; doi:10.1038/srep43205)
Supplement: Supplementary Dataset 1 [file srep43205-s1.doc]

**Soya-cerebroside, an extract of Cordyceps militaris, suppresses monocyte migration and prevents cartilage degradation in inflammatory animal models**

Shan-Chi Liu^1,2#^, Ching-Peng Chiu^3,#^ , Chun-Hao Tsai^4,5^, Chun-Yin Hung^6^, Te-Mao Li^7^, Yang-Chang Wu^8,9,10,11*^ and Chih-Hsin Tang^1,12,13*^

^1^Graduate Institute of Basic Medical Science, China Medical University, Taichung, Taiwan

^2^ Department of Orthopedic Surgery, Taichung Veterans General Hospital, Taichung, Taiwan

^3^Graduate Institute of Natural Products, College of Pharmacy, Kaohsiung Medical University, Kaohsiung, Taiwan

^4^Graduate Institute of Clinical Medical Science, China Medical University, Taichung, Taiwan

^5^Department of Orthopedic Surgery, China Medical University Hospital, Taichung, Taiwan

^6^Department of Orthopaedic Surgery, China Medical University Beigang Hospital, Yun-Lin County, Taiwan

^7^School of Chinese Medicine, China Medical University, Taichung, Taiwan

^8^School of Pharmacy, College of Pharmacy, China Medical University, Taichung, Taiwan

^9^ Research Center for Chinese Herbal Medicine, China Medical University, Taichung, Taiwan

^10^ Chinese Medicine Research and Development Center, China Medical University Hospital, Taiwan

^11^Center of Molecular Medicine, China Medical University Hospital, Taichung, Taiwan

^12^Department of Pharmacology, School of Medicine, China Medical University, Taichung, Taiwan

^13^Department of Biotechnology, College of Health Science, Asia University, Taichung, Taiwan

^#^ **These authors contributed equally to this work**

***Corresponding authors**

Chih-Hsin Tang PhD

Graduate Institute of Basic Medical Science, China Medical University

No. 91, Hsueh-Shih Road, Taichung, Taiwan

Tel: (886)-22052121 Ext. 7726. Fax: (886) 4-22333641.

E-mail: [chtang@mail.cmu.edu.tw](mailto:yfchen@mail.cmu.edu.tw)

**Or** Yang-Chang Wu PhD

College of Pharmacy, China Medical University

E-mail: yachwu@mail.cmu.edu.tw

**Supplementary** **Data**

**
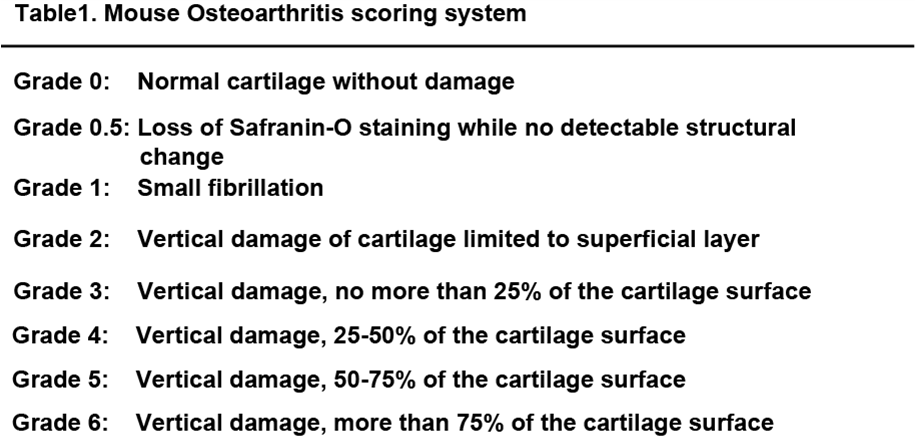
**

**
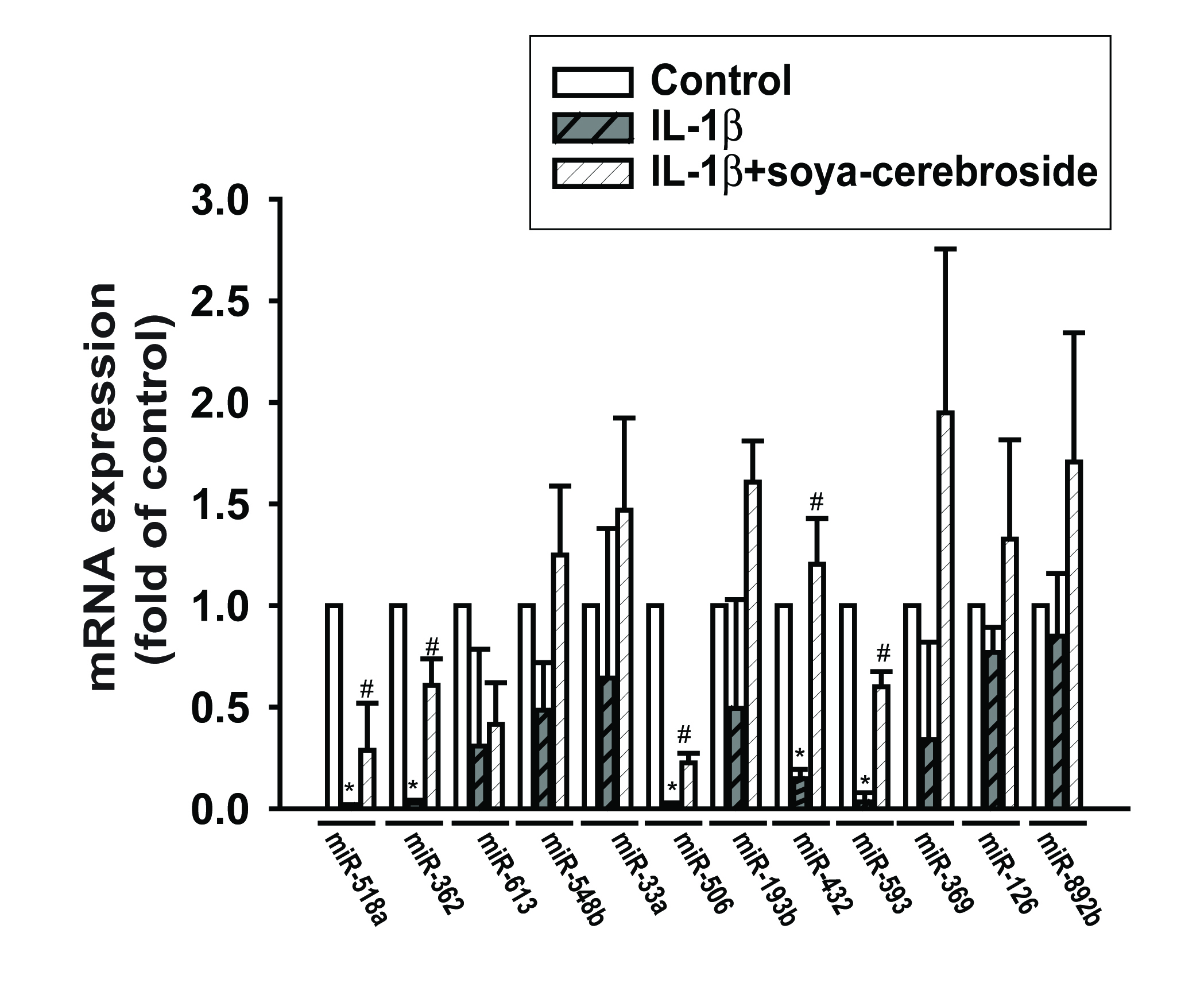
**

**Fig. S1. Using miRNA array to selection the miRNAs expression in soya-cerebroside-treated OASFs.** OASFs were treated with soya-cerebroside in the presence or absence of IL-1β (10 ng/mL) for 24 h, and miRNAs expression were examined by qPCR. Results are expressed as the mean ± standard error of the mean (SEM). *, *p* < 0.05 compared with control; #, *p* < 0.05 compared with IL-1β-treated group.

**
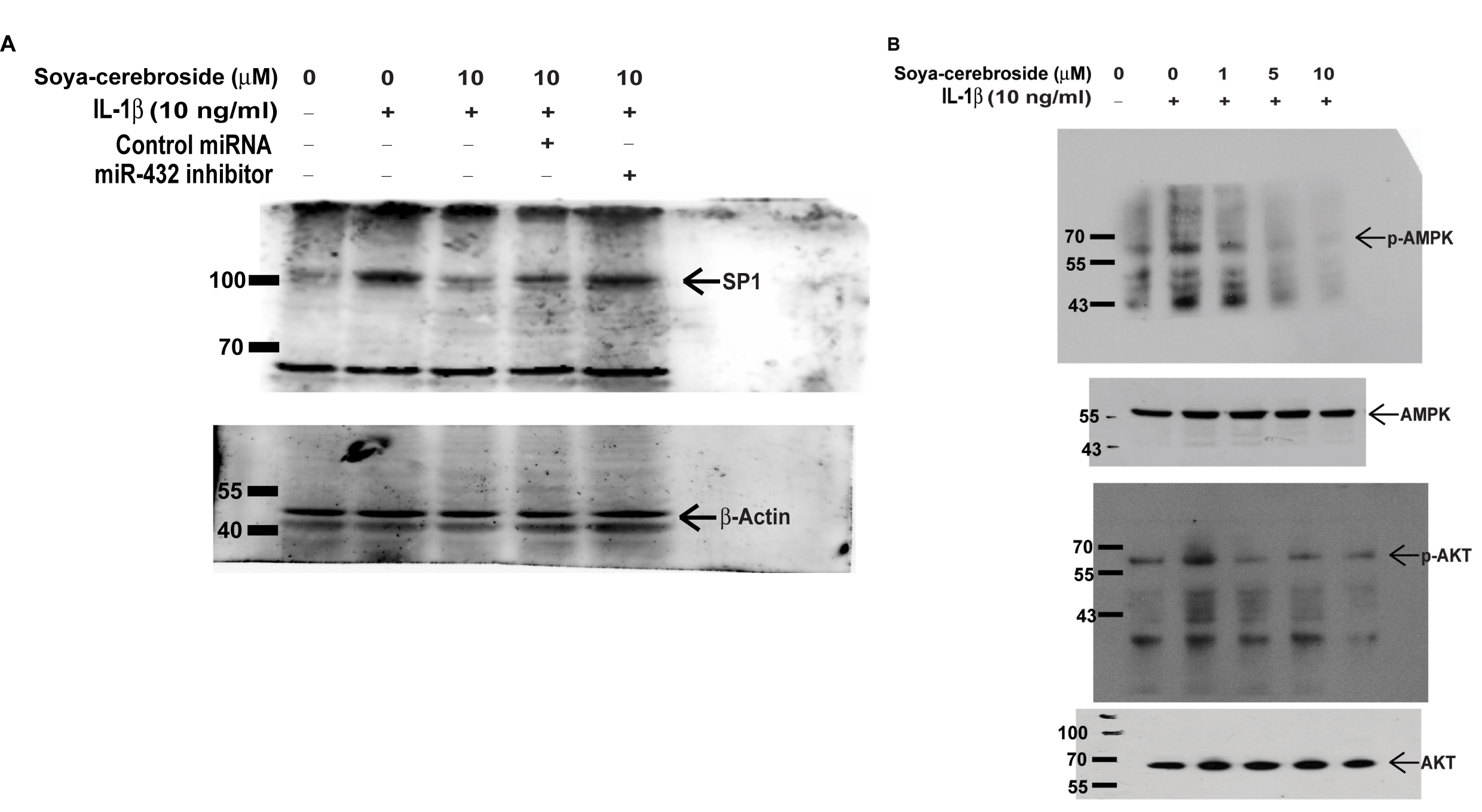
**

### Fig. S2. Uncropped blots for Western blot (A) Full-length immunoblots for detection of SP-1 in OASF, which were transfected with the control miRNA and miR-432 inhibitor prior to treatment with soya-cerebroside (10 μM), followed by IL-1β stimulation. Down panel are depicted full-length blots which have been performed in parallel and stained for actin as loading control. (B) Full-length immunoblots for detection of phospho-AMPK and AKT in OASF which were treated with various soya-cerebroside concentrations in the presence or absence of IL-1β for 1h.
